# Moving Immune Checkpoint Inhibitors to Early Non-Small Cell Lung Cancer: A Narrative Review

**DOI:** 10.3390/cancers14235810

**Published:** 2022-11-25

**Authors:** Giuseppe Viscardi, Fabiana Vitiello, Alberto Servetto, Valerio Gristina, Elio Gregory Pizzutilo, Maria Anna Canciello, Paola Maria Medusa, Fabio Salomone, Gaetano Di Guida, Mariano Mollica, Luigi Aronne, Roberto Scaramuzzi, Filomena Napolitano, Ciro Battiloro, Francesca Caputo, Marina Gilli, Giuseppe Totaro, Carlo Curcio, Danilo Rocco, Vincenzo Montesarchio

**Affiliations:** 1 Medical Oncology, Department of Pneumology and Oncology, AORN Ospedali dei Colli, Via Leonardo Bianchi, 80131 Naples, Italy; 2 Medical Oncology, Department of Precision Medicine, Università degli Studi della Campania “Luigi Vanvitelli”, Via Sergio Pansini 5, 80131 Naples, Italy; 3 Medical Oncology, Department of Clinical Medicine and Surgery, Università degli Studi di Napoli “Federico II”, Via Sergio Pansini 5, 80131 Naples, Italy; 4 Medical Oncology, Department of Surgical, Oncological and Oral Sciences, Università degli Studi di Palermo, Via Liborio Giuffrè 5, 90127 Palermo, Italy; 5 Niguarda Cancer Center, Grande Ospedale Metropolitano Niguarda, Piazza dell’Ospedale Maggiore 3, 20162 Milan, Italy; 6 Departmento of Oncology and Hematology, Università degli Studi di Milano, Via Festa del Perdono 7, 20122 Milan, Italy; 7 Pneumology Unit, Università degli Studi della Campania “Luigi Vanvitelli”, AORN Ospedali dei Colli, Via Leonardo Bianchi, 80131 Naples, Italy; 8 Respiratory Pathophysiology, AORN Ospedali dei Colli, Via Leonardo Bianchi, 80131 Naples, Italy; 9 Thoracic Surgery, Department of General and Specialistic Surgery, AORN Ospedali dei Colli, Via Leonardo Bianchi, 80131 Naples, Italy; 10 Radiotherapy Unit, Istituto Nazionale Tumori IRCCS “Fondazione G. Pascale”, Via Mariano Semmola, 80131 Naples, Italy

**Keywords:** neoadjuvant, adjuvant, immune checkpoint inhibitors, immunotherapy, lung cancer, non-small-cell lung carcinoma (NSCLC)

## Abstract

**Simple Summary:**

Moving from the achievements of immune checkpoint inhibitors in metastatic lung cancer, the great promise of immunotherapy in resectable early-stage is to make a disease, often lethal until now for frequent post-surgery relapses, more curable. This review focuses on role of immunotherapy in early-stage non-small cell lung cancer. We summarize biological rationale, findings from clinical trials of adjuvant and neoadjuvant immune checkpoint inhibitors, questions about biomarkers and choice of endpoints, aiming to provide up-to-date information useful for clinical decision making.

**Abstract:**

Lung cancer is the leading cause of cancer-related death worldwide. Since prognosis of early-stage non-small cell lung cancer (NSCLC) remains dismal for common relapses after curative surgery, considerable efforts are currently focused on bringing immunotherapy into neoadjuvant and adjuvant settings. Previously, perioperative chemotherapy showed only a modest but significative improvement in overall survival. The presence of broad tumor neoantigens load at primary tumor prior to surgery as well as the known immunosuppressive status following resection represent the main rationale for immunotherapy in early disease. Several trials have been conducted in recent years, leading to atezolizumab and nivolumab approval in the adjuvant and neoadjuvant setting, respectively, and perioperative immunotherapy in NSCLC remains a field of active clinical and preclinical investigation. Unanswered questions in perioperative therapy in NSCLC include the optimal sequence and timing of chemotherapy and immunotherapy, the potential of combination strategies, the role of predictive biomarkers for patient selection and the choice of useful endpoints in clinical investigation.

## 1. Introduction

Lung cancer remains the leading cause of cancer-related death worldwide, accounting non-small cell lung cancer (NSCLC) for 85% of all new cases [1]. Currently, only one quarter of patients with NSCLC are diagnosed with early-stage disease and deemed to be eligible for curative-intent surgery [2]. Nevertheless, prognosis remains dismal for the common relapses. With a five-year median follow-up, the percentage of people who have disease recurrence or die after surgery ranges from 45% among patients with stage IB disease to 76% among those with stage III disease, regardless of the use of postoperative chemotherapy [3].

Current international guidelines agree in recommending adjuvant cisplatin-based chemotherapy after complete surgical resection for patients with N1 and N2 disease (stage II to III, according to the 8th TNM classification), basing on a small but significant advantage in 5-year survival [4,5]. The cornerstone Lung Adjuvant Cisplatin Evaluation (LACE) meta-analysis included a total of 4,584 patients from five cisplatin-based adjuvant trials (i.e., IALT, JBR.10, ANITA, ALPI-EORTC and Big Lung Trial) and confirmed the benefit of adjuvant chemotherapy with a 5.4% improvement in survival at 5 years vs. observation (*p* = 0.0043). In this meta-analysis, the effect of a vinorelbine-containing doublet was better in terms of overall survival (OS) and disease-free survival (DFS) compared to other drugs combination (*p* = 0.11 for OS and *p* = 0.07 for DFS) [6].

Preoperative chemotherapy has not been evaluated as extensively as adjuvant. However, the findings of a large individual data meta-analysis suggest that neoadjuvant treatment for resectable disease could represent a valid treatment option, with a magnitude of the survival advantage comparable to that obtained from postoperative chemotherapy, whereas the major pathological response (MPR) rate does not exceed 20% [7]. So far, no evidence of a difference in survival outcomes between the timing of administration of chemotherapy has been obtained [8].

While early diagnoses are expected to increase in the next decades as a result of large scale implementation of low-dose computed tomography (LDCT)-based screening in high risk subjects [9], early NSCLC continues to represent largely an unmet medical need.

In the last decade, immune checkpoint inhibitors (ICIs) have reshaped the treatment scenario of several advanced malignancies. In particular, three immune checkpoint pathways, namely programmed cell death-1 (PD-1), programmed cell death-ligand 1 (PD-L1) and cytotoxic T-lymphocyte antigen 4 CTLA-4, have emerged as target for cancer therapeutics. Indeed, checkpoint blockade using ICIs has the potential to overcome immune evasion and promote an effective immune response against cancer cells [10].

In patients with non-oncogene addicted advanced NSCLC, PD-1/PD-L1 blockade is currently the upfront standard of care as monotherapy, or in combination with chemotherapy, according to PD-L1 expression level [11], whereas treatment with anti PD-L1 durvalumab after curative chemoradiation has demonstrated to prolong survival in stage III unresectable NSCLC [12]. Built on the implementation of ICIs in management of locally advanced and metastatic NSCLC, several studies of neoadjuvant or adjuvant immunotherapy have been carried out in recent years.

Based on the results of two large randomized phase III trials [13,14] in 2022, the Food and Drug Administration (FDA) approved for the first time two immune checkpoint inhibitors for early stage NSCLC: anti PD-L1 atezolizumab for adjuvant treatment of stage II to IIIA resected NSCLC and anti PD-1 nivolumab in combination with platinum-based chemotherapy for preoperative treatment of resectable NSCLC.

This review focuses on the change in the treatment paradigm of early-stage NSCLC due to advent of ICIs starting from biological bases to results of clinical trials and unsolved questions (choice of endpoints and biomarkers, translation of findings to clinical practice).

## 2. Biological Rationale

Recent studies examining the role of ICIs in both neoadjuvant and adjuvant settings rely on strong biological rationales. The main biological argument leading to this line of research, especially in the context of adjuvant setting, is the well-known state of immunosuppression occurring after surgery [15]. Indeed, previous evidence reported various mechanisms arising in postoperative stages, ultimately disrupting the physiological host immune system functions. This immunosuppressive state may favor the growth of tumor cells residing in the tissues surrounding the surgical site [16,17]. Furthermore, various evidence demonstrated that the postoperative alterations of immune system may promote the metastasizing processes [18,19,20]. On the other hand, the changes of the immune environment associated with surgical stress provide a robust rationale for the use of ICIs.

More in detail, the days immediately following surgery are characterized by a local release of soluble factors, such as vascular endothelial growth factor (VEGF), platelet derived growth factor (PDGF), transforming growth factor beta (TGF-β), glucocorticoids, prostaglandins and various cytokines [21,22]. Particularly, a decrease in local secretion of IL-2, IL-12 and interferon gamma (IFN-γ), as well as an increase in levels of IL-6, IL-8, IL-10 and TNF-α have been described (reviewed in [15]). Altogether, this pattern of cytokines contributes to recruitment of T regulatory cells (Treg) and myeloid-derived suppressor cells (MDSC), both leading to an immunosuppressive state [23,24,25,26]. In addition, the cytotoxic activity of Natural Killer (NK) cells, members of the innate response, is strongly impaired [27,28]. Tai and colleagues reported that surgery-induced MDSC impair NK functions through upregulation of arginase 1 (ARG1), IL4Rα and reactive oxygen species (ROS) [29]. Of note, surgical stress can also cause a decline in the number of CD8+ T cells, ultimately reducing the T cell-dependent secretion of IFN-γ, tumor necrosis factor-alfa (TNF-α) and Granzyme B [30,31]. Interestingly, a previous publication reported that PD-1 expression on circulating CD4+, CD8+ T cells, CD14+ monocytes and CD56+ NK cells increased in the days following surgery for NSCLC [32]. As a result, the findings reported above corroborate the use of ICIs, particularly anti-PD-1/PD-L1, as adjuvant treatment for patients with early-stage NSCLC undergoing surgery. The mechanisms determining an immunosuppressive state generally occur within the first days after surgery and may be reversible [17,33]. However, an early postoperative systemic treatment might not be feasible due to unfavorable clinical conditions of patients undergoing major surgery. Next, the ability of anti-PD-1 to fully restore the activity of CD8+ T cells has to be further evaluated [34]. Hence, combination of ICIs with other treatments, such as chemotherapy, radiotherapy or molecularly targeted agents, needs further investigation [35].

High interest in the role of preoperative immunotherapy emerged after demonstration of its superiority compared to postoperative treatment [36,37]. These works were based on the hypothesis that neoadjuvant treatment, despite adjuvant therapies, might be more effective in priming an adaptive immune response, due to the presence of the tumor. Liu and colleagues conducted several in vivo experiments to demonstrate that neoadjuvant immunotherapy promote longer mice survival than adjuvant treatment. More in detail, they employed two models of triple negative breast cancer (TNBC) injected in mammary fat pad genetically engineered mice in which Treg cell population was depleted before or after surgery. Preoperative Treg depletion guaranteed a lower rate of distant metastases and longer survival, compared to postoperative depletion. Similarly, the combination of anti-PD1 and agonistic anti-CD137, to enhance the activity of NK cells [38], promoted longer survival and reduced metastatic burden of TNBC-bearing mice treated before surgery, compared to adjuvant treatment [36]. Finally, the authors revealed that neoadjuvant immunotherapy, despite adjuvant treatment, induced an increase in circulating tumor-specific CD8+ T cells [36]. Interestingly, in a later publication, the same research group also revealed that the timing of surgery after neoadjuvant immunotherapy may influence clinical outcomes [39]. Cascone and colleagues performed similar work, employing the 344SQ murine squamous NSCLC cells that were implanted in the flank of syngeneic mice. These were randomized to receive three doses of IgG, anti-PD1, anti-CTLA4, anti-PD1 + anti-CTLA4 in either a neoadjuvant or adjuvant setting. Preoperative treatment was associated with longer survival than adjuvant therapy, with neoadjuvant combination strategy performing better than single agents [37]. Of course, further investigations are needed to understand the biological and immunological mechanisms governing the differential efficacy of neoadjuvant and adjuvant immunotherapy. Various mechanisms have been described to explain better outcomes of neoadjuvant immunotherapy. First, priming and expansion of primary tumor-specific T cells occurring during neoadjuvant treatment are more effective than adjuvant immunotherapy, with the expansion of a higher number of tumor-resident T cell clones in peripheral blood [40,41]. Of note, the adaptive immunity triggered by neoadjuvant immunotherapy promotes expansion and activation of not only effector cytotoxic T cells but also memory T cells that can contribute to eradicate residual tumor cells after surgery, preventing relapse of the disease [42]. Recent evidence further clarified that neoadjuvant immunotherapy promotes both local and systemic T cell responses [43]. In addition to T cell response within the primary site, tumor-draining lymph nodes (TDLN) are crucial sites for antigen presentation to tumor-specific T cells [44]. Multiple evidence has demonstrated that tumor-specific antigens are carried by dendritic cells to TDLN and presented to CD8+ T cells [45,46]. Based on these premises, the immune response following adjuvant treatments might be less effective due to removal of TDLN during surgery of primary NSCLC.

While the combination of cytotoxic agents and ICIs is known to be effective for the treatment of stage IV NSCLC, its role in the neo/adjuvant setting is still debated. First, chemotherapy is generally considered as immunosuppressive leading to myelosuppression and leukocytopenia, therefore theoretically inappropriate for combination with ICIs [47,48,49]. However, chemotherapy can cause various multiple effects on the different components of the immune response. Indeed, it can cause selective downregulation of Treg and MDSC, with a reduced effect on other CD4+ and CD8+ cells [50,51]. The canonical genotoxicity of chemotherapy and radiotherapy leads to generation of neo-antigens expressed by cancer cells, thereby potentially stimulating T cell response when these neo-antigens are expressed by antigen presenting cells (APC) in lymph nodes. However, the association between tumor “antigenicity” and sensitivity to immunotherapy has to be further clarified [52]. More likely, the benefit of combining chemo/radiotherapy with immunotherapy relies on the ability of genotoxic treatments to induce “adjuvanticity” of the immune system, that is the capability to strengthen the induction of an adaptive immunity, leading to an immunogenic tumor cell death (ICD). Indeed, cytotoxic agents, as well as radiotherapy, can induce the expression of damage-associated molecular patterns (DAMPs), such as calreticulin, extracellular ATP and high mobility group box 1 (HMGB1) that can be recognized by macrophages and dendritic cells (DCs), ultimately triggering and reinforcing the adaptive immunity [53]. Of note, different clinically available cytotoxic agents induce distinct effects on immune response and ICD profiles [48]. Hence, clinical trials are mandatory to establish the best combination strategy for neo/adjuvant treatment in NSCLC.

## 3. Neoadjuvant Immunotherapy

Biological rationale translated into clinical research starting from a pilot study by Forde et al. In this single group study, two preoperative doses of PD-1 inhibitor nivolumab were administered 4 and 2 weeks prior to planned surgery in early resectable (stage I to IIIA) NSCLC achieving a MPR and a pathological complete response (pCR) in 9 (45%) and 2 (10%) of 20 of resected tumors, respectively. Twenty (95%) of 21 eligible patients underwent complete resection at a median interval time of 18 days from second drug administration. No treatment-related surgical delays occurred, whereas the only one grade 3 treatment-related adverse event (TRAE) observed (pneumonia) did not prevent the planned surgery, also performed without complications. Radiologically, only one (5%) progression of disease (PD) occurred whereas 18 (86%) patients had stable disease (SD). Moreover, a recurrence-free survival (RFS) of 69% at 24 months has been reported [41].

Subsequent single-agent trials with different PD-1 or PD-L1 inhibitors confirmed these findings. In these studies, MPR ranged from 14 to 40.5% and pCR reached 16% [54,55,56,57,58].

Moving from the preclinical rationale, a dual-immune checkpoint blockade has been tested to ameliorate these pivotal results.

Although the study was not powered for MPR comparison between arms, Cascone et al. reported from a phase II NEOSTAR trial that the addition of ipilimumab to three doses of neoadjuvant nivolumab increased MPR from 24 to 38% and pCR from 9 to 29%. Forty-four patients with resectable NSCLC (stage I to IIIA N2 single station) were enrolled and trial met prespecified efficacy boundary. As a biological counterpart, greater proliferation of different T cell subsets and T-cell receptor (TCR) homology was observed after dual immunotherapy compared with nivolumab alone [59].

Likewise, an amendment to the study of Forde and colleagues allowed the inclusion of a new arm investigating neoadjuvant nivolumab and ipilimumab. Disappointingly, the trial was prematurely stopped after 9 of 15 planned patients were enrolled. Indeed, grade ≥3 TRAEs were observed in three (33%) patients and only six (66%) patients underwent surgery, of whom two with pathologic complete response (pCR), whereas three patients experienced biopsy-proven disease progression. Interestingly, two of them carried KRAS/STK-11/KEAP-1 co-mutations [60].

Other strategies related to the object of investigation encompass the combination of anti PD-1/PD-L1 with novel immunotherapies or chemotherapy.

The phase II NeoCOAST platform trial highlighted feasibility and activity of a single cycle neoadjuvant durvalumab combined with oleclumab (anti CD-73), monalizumab (anti-NKG2A) or danvatirsen (an anti-STAT3 antisense oligonucleotide). The study was not formally powered to compare arms, but MPR investigator-assessed rates ranged from 19% (oleclumab arm) to 31.3% (danvatirsen group). Whole RNA sequencing was performed on paired baseline and post-surgery tissue, demonstrating a greater increase of expression of genes associated with NK and CD8 T cells, cytotoxicity, tertiary lymphoid structures and lymphocyte recruitment with durva+mona or durva+ole than with durvalumab alone [61]. Building on these results, a phase III follow-up randomized clinical trial, NeoCOAST-2, is currently enrolling [62].

Furthermore, different chemotherapy backbones have been explored in association with anti PD-1/PD-L1.

The rate of MPR and pCR exceed 80% and 60%, respectively, in a single-arm phase II trial done in 18 hospitals in Spain and investigating a peri-operative approach of neoadjuvant carboplatin + paclitaxel + nivolumab for three cycles and one year of adjuvant nivolumab. Only patients with stage IIIA disease were included, of which about half had multiple N2 disease. Forty-one (89%) of 46 enrolled patients achieved complete resection, and no disease progression occurred during the treatment. The 24-months progression-free survival (PFS) reached almost 80%; OS at 3 years in the intention-to-treat (ITT) population was 81.9% [63].

In another single-arm phase 2 trial involving three institutions in the United States of America (USA), 30 patients were treated with up to four cycles of neoadjuvant nab-paclitaxel + atezolizumab for stage IB-IIIA NSCLC. In this case, a R0 resection was performed in 87% of patients, with pCR and MPR of 33% and 57%, respectively. [64]

More recently, the first survival data from a follow-up randomized trial of NADIM II were released.

In NADIM II patients with potentially resectable stage IIIA-B NSCLC, without Epidermal Growth Factor Receptor (EGFR) or Anaplastic Lymphoma Kinase (ALK)-driven disease, were randomized to receive carboplatin paclitaxel ± nivolumab up to three cycles in neoadjuvant phase, then nivolumab or placebo for six months after surgery. Of 86 enrolled patients, about one third had N2 multiple station disease in both arms. The primary endpoint was reached, increasing the percentage of patients with pCR from 6.8 to 36.2%, compared to chemotherapy alone (RR 5.25; 99% CI 1.32–20.87; *p* = 0.0071). Looking at secondary endpoints, at a median follow-up of 21.9 months, the PFS rate at 24 months was improved from 52.6% to 67.3% (HR 0.56; 95% CI 0.28–1.15; *p* = 0.117), and OS from 64.8% to 85.3% (HR 0.37; 95% CI 0.14–0.93; *p* = 0.003). Moreover, the surgery rate was increased from 65.0 to 92.5% (OR 6.60; 95% CI, 1.67–26.02, *p* = 0.007). As shown in an exploratory subgroup analysis, PD-L1 expression (≥1%) seemed to identify patients with improved PFS (HR 0.26; 95%CI 0.08–0.77; *p* = 0.015) in the experimental arm [65].

Lastly, neoadjuvant combination of platinum-doublet chemotherapy and nivolumab was approved from FDA on March 2022 on the basis of findings of randomized phase III trial CheckMate 816. In this study, 358 patients diagnosed with resectable stage IB-IIIA NSCLC, lacking sensitizing mutations of EGFR and ALK, were randomized to receive histology-based chemotherapy ± nivolumab for three cycles prior to surgery. At baseline, more than 60% of patients had IIIA disease and PD-L1 expression was found in about half of cases. Both pCR and event-free survival (EFS) according to blinded independent central review (BICR) were primary endpoints. pCR was improved from 2.2% (95% CI 0.6–5.6) with chemotherapy alone to 24.0% (95% CI 18.0–31.0) with nivolumab plus chemotherapy (OR 13.94; 99% CI 3.49–55.75; *p* < 0.001),and was observed regardless of the initial stage and radiologic downstaging. With a minimum follow-up of 21 months, median EFS was improved from 20.8 (95% CI 14.0–26.7) to 31.6 months (95% CI, 30.2-NR), favoring experimental arm. According to a subgroup analysis it can be hypothesized that EFS advantage for immunotherapy containing arm is driven from the IIIA stage (HR for EFS 0.54; 95% CI, 0.37–0.80) rather than the IB-II stage (HR 0.87, 95% CI 0.48–1.56). However, the OS data are still processing. Definitive surgery was performed in 83% of patients with nivo + chemo (*n* = 149) vs. 75% with chemo (*n* = 135), without an increase in any-grade and grade 3–4 surgery-related adverse events (AEs). Despite the positive results, 17% of patients in chemoimmunotherapy arm and 25% in chemotherapy group did not receive surgery, because of disease progression, adverse events, patient refusal, unresectability and poor functional reserve [14].

Neoadjuvant immunotherapy trials are summarized in Table 1 and Table 2.

## 4. Adjuvant Immunotherapy

Looking at immunotherapy as an adjuvant strategy after curative-intent surgery for early NSCLC, atezolizumab was the first ICI to receive FDA approval as adjuvant therapy for PD-L1 expressing stage II to IIIA NSCLC following surgical resection and completion of adjuvant cisplatin-based chemotherapy, based on the results of phase III trial IMpower 010. In this study, PD-L1 expression was assessed using the SP263 immunohistochemistry (IHC) assay. After complete resection for stage IB-IIIA NSCLC, 1280 patients were enrolled, then 1005 of them were randomized at the end of adjuvant platinum-based chemotherapy to receive adjuvant atezolizumab (for 16 cycles or 1 year) or best supportive care (BSC). In total, 41.1% of patients had IIIA-resected tumor. The primary endpoint was investigator-assessed disease-free survival (DFS) evaluated in the stage II-IIIA PD-L1 positive population, in all the patients with stage II-IIIA, and in the ITT population including all randomized patients with stage IB-IIIA NSCLC. Primary and secondary endpoint of OS in ITT population were tested hierarchically. Prespecified subgroup analyses included DFS and OS evaluation according to PD-L1 expression.

The study met its primary endpoint, improving adjuvant atezolizumab DFS in resected PD-L1 positive stage II-IIIA (median DFS NR v 35.3 months; HR 0.66; 95% CI, 0.50 to 0.88; *p* = 0.004). As a secondary endpoint, the benefit appeared to be most pronounced in patients with PD-L1 expression > 50% (median DFS NE v 35.7 months; HR 0.43; 95% CI 0.27–0.68).

At first interim analysis of OS conducted at 45.3 months of median follow-up, IMpower010 showed a trend in favor of atezolizumab in the PD-L1 TC > 1% stage II-IIIA population (HR 0.71, 95% CI 0.49–1.03) but not in the all randomized stage II-IIIA (HR 0.95, 95% CI 0.74–1.24) or intent-to-treat population (HR 0.995, 95% CI 0.78–1.28). As these analyses were driven by subgroups and more mature data are still awaiting, there remains substantial debate over which patient populations should be selected for adjuvant checkpoint inhibitors.

In the Impower 010 any-grade AEs were reported in 92.5% of patients in the atezolizumab arm and 70.9% in the best supportive care (BSC) arm, of which 67.9% and 0.0%, respectively, were treatment-related. Moreover, 22.0% and 11.5% of AEs in either arm were grade 3/4, 10.7% and 0.0% of which were treatment related. Investigators also reported that 7.5% of patients in the atezolizumab arm and 0.0% of those in the BSC arm experienced serious treatment-related AEs, and 1.8% and 0.6% had grade 5 AEs, respectively. These findings showed no new safety signals [13].

Additionally, an interim analysis of a trial evaluating adjuvant pembrolizumab has recently been reported. The PEARLS/KEYNOTE-091 trial evaluated one year of adjuvant pembrolizumab compared with placebo in patients with any PD-L1 expression and IB (T ≥ 4 cm) to IIIA NSCLC (per AJCC 7th edition) following surgical resection and chemotherapy (which was strongly recommended for stage II and II and considered for stage IB disease, but not required). In total, 1177 patients were randomized, of whom 28.8% resected for stage IIIA tumor. Dual primary end points were DFS in the all-comers and PD-L1 tumor proportion score (TPS) > 50% population. PD-L1 was determined using the PD-L1 IHC 22C3 assay, and the TPS. The primary endpoint of DFS was significantly improved with pembro in the all-comers population (median 53.6 vs. 42.0 mo; HR 0.76; 95% CI 0.63–0.91; *p* = 0.0014). Surprisingly, at interim analysis, the significance boundary was not crossed for the TPS ≥ 50% population (median DFS NR in either arm; HR 0.82; 95% CI 0.57–1.18; *p* = 0.14). An exploratory subgroup analysis suggested that the DFS benefit may have been driven by the TPS PD-L1 1–49% group. The pembro safety profile was as expected; AEs were grade ≥ 3 in 34.1% of pts in the pembro arm vs. 25.8% in the placebo arm and led to discontinuation in 19.8% vs. 5.9%; treatment-related Aes led to death in 0.7% vs. 0% [66].

Finally, results of additional pivotal phase III trials evaluating adjuvant use of durvalumab (BR31, NCT02273375) [67] and nivolumab (ANVIL, NCT02595944) are expected [68].

Table 3 and Table 4 provide a summary of adjuvant immunotherapy trials in NSCLC.

## 5. Choice of Endpoints

As mentioned above, the choice of meaningful outcomes and validation of surrogate endpoints represents a critical aspect in conducting perioperative trials, both for clinical relevance of findings and regulatory aspects. Traditionally, early-stage disease trials using overall survival as primary endpoint require long time to complete, potentially limiting transferability to clinical practice and increasing costs.

Hence, neoadjuvant immunotherapy trials focused on endpoints of activity and safety, including appearance of TRAEs and feasibility of surgery.

In the chemotherapy era, MPR, defined as ≤10% residual viable tumor cells, has been proposed as a surrogate endpoint for the neoadjuvant approach, due to reported association with a magnitude of treatment benefits and prediction of survival improvement [69]. Furthermore, MPR rates with ICI-containing regimens are higher than those observed with chemotherapy alone.

Instead, the absence of residual viable tumor cells in both primary tumor and sampled lymph nodes defines the occurrence of pCR and offers the advantage to be more reproducible than MPR as outcome [70]. Both Checkmate 816 and NADIM II reported pCR as primary endpoint. Furthermore, in the former, a trend towards improvement of EFS was observed in patients with pCR compared to those without (HR for disease progression, recurrence or death 0.84, 95% CI 0.61–0.17) [14]. Similarly, in an exploratory analysis of the latter at time of report none of the patients achieving a pCR has progressed or deceased at time of evaluation [65].

Significantly, to enhance reproducibility between pathologists, recently, the international association for study of lung cancer (IASLC) has released detailed recommendations addressing the need for standardized processing and evaluation of surgical specimens after neoadjuvant treatment [71].

Radiologic imaging evaluation after neoadjuvant immunotherapy represents a further challenge because it does not necessarily reflect the therapeutic effect on cancer cells. This may be due to inflammatory stromal and fibrotic modifications that resemble viable cancer cells [72]. Indeed, pseudo progression also can be observed. Thus, authors of NADIM trial underlined that pCR was achieved by three (33%) of nine patients with SD, in addition to 22 (73%) of 30 patients with a partial response (PR) [73]. Elsewhere it is reported how the rate of MPR/pCR generally exceeds that of overall response rate (ORR). The use of ^18^F-fluorodeoxyglucose positron emission tomography-computed tomography (^18^F-FDG PET-CT) could have a stronger correlation with pathologic response than traditional CT scan aiding to overcome some limitations of a morphological evaluation [74].

In addition to radiologic and pathologic outcomes, surgical issue can be carefully evaluated when offering neoadjuvant treatment to patients with resectable NSCLC, due to risk of toxicity or progression of disease potentially delaying or hampering surgery. In the CheckMate 816 trial, lower rates of pneumonectomy (17% vs. 25%) were observed in the chemo-immunotherapy arm compared with chemotherapy alone. Adverse events cause delays of surgery in six vs. nine patients, respectively. No increase in median duration of surgery and length of hospitalization was observed. The conversion from minimally invasive to thoracotomy surgery was necessary in 11% and 16% of patients randomized to experimental and standard arm, respectively [75]. These findings were confirmed in a large meta-analysis encompassing 16 trials of neoadjuvant (chemo)-immunotherapy [76].

The evaluation of circulating tumor DNA (ctDNA) has the potential to outperform Response and Evaluation Criteria in Solid Tumors (RECIST)-based criteria on predict survival in neoadjuvant setting. In the NADIM trial, baseline low ctDNA levels (<1% mutant allele frequency) as assessed by Oncomine Pan-Cancer Cell-Free Assay predicted OS (HR, 0.07; 95% CI, 0.01 to 0.39) [77]. Moreover, ctDNA clearance during neoadjuvant treatment may be an early predictor of favorable outcomes, as shown both in the NADIM and CheckMate 816 trials. In the Spanish trial, undetectable ctDNA levels after neoadjuvant treatment were associated with better OS (HR, 0.04; 95% CI, 0.00 to 0.55, respectively). Conversely, CT scan assessed radiological response was not associated with survival [63]. In the CheckMate 816 trial, ctDNA clearance was more frequent with chemo-immunotherapy compared to standard treatment (56%; 95% CI, 40 to 71 vs. 35%; 95% CI, 21 to 51) and this event seems to correlate with better EFS in both arms [14].

Regarding the adjuvant setting, longer-term follow up is needed to determine whether these therapies merely delay recurrence or can improve OS and increase the cure rate following surgical resection, as both Impower 010 and PEARLS elected DFS as primary endpoint and mature OS data are still awaiting. If the value of DFS as surrogate endpoint of OS in adjuvant has been demonstrated in different types of solid tumors [78,79], it can be argued with postoperative use of tyrosine kinase or immune checkpoint inhibitors. Meanwhile, the significancy of DFS as surrogate endpoint relies on the amplitude of its benefit as the likelihood to improve OS in adjuvant NSCLC relates to the magnitude of DFS advantage. Furthermore, remaining longer in the “curative intent setting” and delaying burden symptoms due to disease recurrence can be valuable from the patient’s perspective.

## 6. Role of Biomarkers

Trials investigating immunotherapy use in early-stage disease have established a critical therapeutic transformation, and investigators have begun to focus on how to optimize these therapies on the basis of clinical features and tumor biomarkers (Table 5).

In the seminal paper of Forde et al., both PD-L1 negative and PD-L1 positive patients achieved MPR [41], unlike the LCMC3 trial (29% of MPR in PD-L1 positive vs. 16% in PD-L1negative) [80]. Both in NADIM and NADIM-II trials, patients with pCR had higher PD-L1 TPS [63,65]. Lastly, signals of greater benefit from the addition of immunotherapy to standard neoadjuvant chemotherapy in PD-L1-positive patients arise from a subgroup analysis of CheckMate 816 (HR for EFS in PD-L1 negative: 0.85 vs. 0.24 in PD-L1 ≥ 50%) [14]. Other explored biomarkers in the neoadjuvant setting include tumor mutational burden (TMB), T-cell receptor repertoire and immunophenotype [81].

Additionally, in the adjuvant setting, whether certain populations would benefit more from a specific approach will remain a topic of debate. In fact, in IMpower010, PD-L1 negative tumors did not appear to benefit from adjuvant atezolizumab (HR 0.97; 95% CI 0.72–1.31) and the greatest benefit was observed in tumors with PD-L1 expression ≥ 50%. As reported above, in the PEARLS trial, a DFS benefit was observed in the overall patient population, and not observed in the subset of patients with a tumor with TPS PD-L1 ≥ 50%.

Adjuvant immunotherapy trials included a small percentage of patients with EGFR- or ALK-driven disease who appeared to have a limited benefit from this approach. No DFS advantage for atezo vs. BSC was reported in IMpower010 for 109 stage II-IIIA patients harboring sensitizing EGFR mutations (HR 0·99, 95% CI 0·60–1·62), whereas HR in the subgroup of EGFR-mutated PD-L1+ patients was 0·57 (95% CI 0·26–1·24) [13]. Thus, they are not considered appropriate candidates for adjuvant immunotherapy and targeted options should be prioritized in this setting (e.g., osimertinib in resected tumors harboring EGFR mutations).

With regard to neoadjuvant trials, patients harboring EGFR and ALK alterations were excluded from NADIM II and Checkmate 816 [14,65]. In the LCMC3 study, among 16 EGFR/ALK positive tumors at resection, no one demonstrated a radiographical response or MPR [58]. Conversely, in NeoCOAST, among patients with MPR, two had EGFR mutations (both in durva + ole arm) [61].

Future research efforts will focus on minimal residual disease (MRD) to prioritize patient groups most likely to benefit from checkpoint inhibitors (or target therapy) in the early-stage setting. As mentioned, ctDNA analysis can identify residual/recurrent disease prior to radiological progression in resected patients [82] and can be used to guide immune-based therapy for patients with NSCLC [79]

In the ITT population from ImPower 010, 600 patients (60%) were evaluable for ctDNA analysis and a positive relationship was observed between ctDNA and disease stage, nodal status and EGFR mutations. Post-surgery ctDNA positivity was a poor prognostic factor for DFS in all evaluable stage II-IIIA patients (HR = 0.69, 95% CI: 0.53, 0.89), whereas HR for DFS in the atezo arm vs. BSC was 0.72 (95% CI 0.52, 1.00) and 0.61 (95% CI 0.39, 0.94), respectively in ctDNA positive and ctDNA negativepatients [83].

Finally, the intensification of adjuvant treatment according to MRD through ctDNA analysis has led to design of MERMAID-1, in which resected patients with MRD+ post-surgery were randomized to receive chemotherapy + durvalumab or placebo (until 1 year) [84], and MERMAID-2 trial, randomizing MRD+ patients after curative-intent therapy to durvalumab or placebo [85], whose results are awaiting.

## 7. Conclusions

In the last decade, ICIs have become an essential component of front-line systemic treatment of advanced non-oncogene addicted NSCLC, achieving a doubled rate of long-term survivors among patients strongly expressing PD-L1 who received pembrolizumab (5-year OS 31.9%, 95% CI 24.5–39.5) vs. standard chemotherapy (5-year OS 16.3%, 95% CI 10.6–23.0) from the breakthrough Keynote-024 trial [86].

However, results from clinical trials and daily practice suggest that the majority of unselected patients with metastatic lung cancer are refractory to ICIs, or develop acquired resistance, for reasons that are not fully understood [87].

So, cancer surgery remains the most effective single modality for curing patients. The great promise of immunotherapy bringing in earliest stages of NSCLC is to make an often lethal disease more curable for frequent post-surgery relapses, by precociously priming an elevated and sustained peripheral tumor-specific immune response. Well-designed trials in this setting should help clinicians to define new algorithms of treatment, providing some evidence for the selection of patients to prioritize a neoadjuvant or adjuvant approach and the optimal use of biomarkers. A longer follow-up of current studies is also needed to confirm the translation of surrogate endpoint benefits on survival.

The potential advantages of preoperative immunotherapy include better compliance and assessment of treatment efficacy prior to surgery, treatment of micro-metastases at earliest time point, availability of pre- and post-surgical specimens for translational studies. Moreover, neoadjuvant ICIs may elicit a more sustained immune response for the presence of broad tumor neoantigens load at primary tumor and an intact functioning of T cells [88]. Adjuvant immunotherapy has the potential to avoid delaying or hampering of potentially curative surgery, but clinicians would need biomarkers of risk relapse (or immunotherapy benefit) to avoid a potential overtreatment of patients already cured by surgery alone.

Further areas of uncertainty regard the optimal duration of therapy, criteria for de-escalation or escalation after neoadjuvant immunotherapy and surgery, and the role of chemotherapy and radiation.

As trials comparing neoadjuvant versus adjuvant immunotherapy in resectable NSCLC are lacking, some evidence supporting preoperative treatment come from preclinical [36] and clinical investigation [89] conducted in others tumor types. In the S1801 trial, patients with stage IIIB-IV operable melanoma were treated with three doses of pembrolizumab leading up to surgery, then an additional 15 doses following surgery, achieved better EFS than those receiving upfront surgery followed by 18 cycles of the same anti PD-1 drug [89].

Based only on available data, one can speculate that a preoperative approach would be preferable in high-risk patients or more advanced stages, whereas adjuvant therapy could take its place when delaying surgery is not advised.

Moreover, the introduction of osimertinib in postoperative treatment of EGFR addicted NSCLC [90] as well as the different targeted therapies under evaluation in early-stage disease [91] make mandatory a precocious molecular profiling in early-stage NSCLC to inform decisions on adjuvant and neoadjuvant strategies.

Finally, further issues surround the optimal treatment of stage IIIA-N2 as well as borderline (or potentially resectable) disease, being at present the definition of resectability is largely dependent on local expertise [92] and evidence about the benefits of surgery is lacking [93]. In the immunotherapy era, trials of surgical vs. non-surgical strategies are needed to establish the best approach (definitive chemoradiation followed by immunotherapy [94] or perioperative strategy) for this heterogeneous group of patients.

Certainly, while clinical complexity is set to increase, it is strongly recommended that all patients with early-stage NSCLC are routinely evaluated in a multidisciplinary meeting to define the ideal treatment strategy for each patient.

## Figures and Tables

**Table 1:** Trials of neoadjuvant immunotherapy in resectable early NSCLC (results available).

Trial	No. of Pts	Stage	Phase	Study Design	pCR	ORR	PFS or EFS	mOS	≥G3 AEs	R0 Surgery
CheckMate 816	358	IB-IIIA	3	nivo + CT/CT -> surgery	24%/2.2%	10%	mEFS = 31.6 m vs. 20.8 m	NR	33.5%/36.9%	83%/78%
IONESCO	46	IB > 4 cm-IIIA	2	durva × 3 c -> surgery	7%	9%	NR	NR	NA	89%
LCMC3	181	IB-IIIB	2	atezo × 2 c -> surgery -> atezo × 12 m	7%	7%	At 1yr: 85%	NR	9%	92%
NADIM	46	IIIA	2	nivo + CT × 3 c-> surgery -> nivo × 12 m	4%	76%	At 2yr: 77%	NR	30%	89%
NADIM-II	87	IIIA-IIIB	2	nivo + CT/CT × 3 c -> surgery -> nivo/FU × 6 m	36.2%/6.8%	74%/48%	mPFS: NR vs. 18.3 m	NR	24%/10%	NA
NCT02259621	15	IB-IIIA	1b/2	nivo + ipi ×1 c -> surgery	30%	11%	NA	NA	33%	NA
NCT0271638	30	IB-IIIA	2	atezo + CT × 2 c -> surgery	33%	63%	mPFS: 17.9 m	NR	71%	87%
NCT02904954	60	I-IIIA	2	durva + SBRT × 2 c/durva -> surgery	26.6%/6.7%	46.7%/3.3%	NR	NA	20%/17%	83%/77%
NeoCOAST				durva, durva + ole, durva + mona, durva + danva	3.7%, 9.5%, 10.0%, 12.5%	7.4%, 4.8%, 15.0%, 6.3%	NR	NR	0%, 4.8%, 0%, 6.3%	NR
NEOSTAR	44	I-IIIA	2	nivo + ipi/nivo × 1 c -> surgery	29%/9%	22%/19%	NR	NR	13%/10%	100%
NeoTAP01	33	III	2	tori + CT × 3 c -> surgery	50%	NA	NR	NR	18.1%	87.9%
PRINCEPS	30	I-IIIA	2	atezo × 1 c -> surgery	0%	7%	NA	NA	0%	97%
SAKK 16/14	68	IIIA	2	CT × 3 c -> durva × 2 c -> surgery -> durva × 12 m	18%	NA	NR	NR	88%	93%

List of abbreviations: AEs: Adverse Events; atezo: atezolizumab; c: cycles; CRT: Combined Chemo-Radiotherapic Treatment; CT: chemotherapy; danva: danvatirsen; durva: durvalumab; EFS: Event Free Survival; FU: follow-up; ipi: ipilimumab; m: months; mona: monalizumab; mOS: median Overall Survival; NA: Not Available; nivo: nivolumab; NR: Not Reached; ole: oleclumab; ORR: Objective Response Rate; pCR: pathologic Complete Response; PFS: Progression Free Survival; sinti: sintilimab; tori: toripalimab.

**Table 2:** Trials of neoadjuvant immunotherapy in resectable NSCLC (ongoing or awaiting results).

Trial	No. of Pts	Stage	Phase	Study Design
AEGEN	800	IB-IIIA	3	durva/placebo + CT × 4 c -> surgery -> durva/placebo × 12 c
CANOPY-N	110	IB-IIIA	2	canaki/pembro/canaki + pembro × 2 c -> surgery
CheckMate 77T	452	IIA-IIIB	3	nivo/placebo + CT × 4 c -> surgery -> nivo/placebo × 12 m
IMpower030	450	II-IIIA	3	atezo/placebo + CT x 4 c -> surgery -> atezo × 16 c/BSC
KEYNOTE-671	786	IB-IIIA	3	pembro/placebo + CT × 4 c -> surgery -> pembro/placebo × 13 c
NeoCOAST-2	140	II-IIIA	2	durva + ole/mona + CT × 4 c -> surgery -> durva + ole/mona

List of abbreviations: atezo: atezolizumab BSC: Best Supportive Care; c: cycles; canaki: canakinumab; CT: chemotherapy; durva: durvalumab; FU: follow-up; m: months; mona: monalizumab; nivo: nivolumab; ole: oleclumab; pembro: pembrolizumab.

**Table 3:** Trials of adjuvant immunotherapy in resectable early NSCLC (results available).

Trial	No. of Pts	Stage	Phase	Study Design	DFS	mOS	≥G3 AEs
IMpower010	1280	IB-IIIA	3	surgery -> aCT -> atezo × 16 c/BSC	NR/37.2 m	NR	21.8%/11.5%
KEYNOTE-091	1177	IB-IIIA	3	surgery -> aCT -> pembro/placebo ×12 m	53.6/42.0 m.	NR	34.1%/25.8%

List of abbreviations: aCT: adjuvant chemotherapy; AEs: Adverse Events; atezo: atezolizumab; BSC: Best Supportive Care; c: cycles; DFS: Disease Free Survival; m: months; pembro: pembrolizumab; mOS: median Overall Survival; NA: Not Available; NR: Not Reached.

**Table 4:** Trials of adjuvant immunotherapy in resectable early NSCLC (ongoing or results awaiting).

Trial	No. of Pts	Stage	Phase	Study Design
ALCHEMIST chemo-IO	1263	IB-IIIA	3	surgery -> (aCT ± PORT -> observation)/(aCT ± PORT -> pembrox 16 c)/(aCT + pembro c1,3 ± PORT -> pembro × 12 c)
ANVIL	903	IB-IIIA	3	surgery -> aCT -> nivo/BSC × 12 m
BR.031	1360	IB-IIIA	3	surgery -> aCT -> durva/placebo × 12 m
CANOPY-A	1500	II-IIIA/IIIB	3	surgery -> aCT -> canaki/placebo × 18 c
MERMAID-1	332	II-III	3	surgery -> MRD testing -> aCT + durva/placebo × 4 c -> Durva/Placebo × 10 c
MERMAID-2	284	II-III	3	surgery -> aCT -> MRD+ -> durva/placebo × 24 m
NADIM-ADJUVANT	210	IB-IIIA	3	surgery -> aCT ±nivo x4 c -> nivo x 6 c/observation

List of abbreviations: aCT: adjuvant chemotherapy; BSC: Best Supportive Care; c: cycles; canaki: canakinumab; durva: durvalumab; m: months; MRD: minimal residual disease; nivo: nivolumab; pembro: pembrolizumab; PORT: post-operative radiotherapy

**Table 5:** PD-L1 as biomarker for perioperative immunotherapy in NSCLC.

Biomarker	Neoadjuvant Trials	Adjuvant Trials
PD-L1	pCR in NADIM II: <1%, 14.3%; 1%–49%, 41.7%; ≥50%, 61.1%, *p* = 0.008 pCR in Checkmate 816, experimental vs. control arm (95% CI): <1%, 16.7% (9.2–26.8) vs. 2.6% (0.3–9.1); 1–49%, 23.5% (12.8–37.5) vs. 0% (0–7.5); ≥50%, 44.7 (28.6–61.7) vs. 4.8 (0.6–16.2)	HR for DFS (95% CI) in Impower 010: <1%, 0.97 (0.72–1.31); 1–49%, 0.87 (0.60–1.26); ≥50%, 0.43 (0.27–0–68) HR for DFS (95% CI) in Keynote 091/PEARLS: <1%, 0.78 (0.58–1.03); 1–49%, 0.67 (0.48.0.92); ≥50%, 0.82 (0.57–1.18)

List of abbreviations: confidence interval (CI), disease-free survival (DFS), hazard ratio (HR), pathologic complete response (pCR), programmed death ligand 1 (PD-L1).
